# Development of a markerless tool for targeted chromosome modification in the thermophilic and methylotrophic bacterium *Bacillus methanolicus*

**DOI:** 10.1186/s12934-025-02880-0

**Published:** 2025-12-10

**Authors:** Marta Irla, Luciana Fernandes Brito, Jesper Langlo, Carsten Wohlers, Leonie Benninghaus, Chantel Heid, Volker F. Wendisch, Jochen Schmid, Trygve Brautaset

**Affiliations:** 1 Department of Biological and Chemical Engineering, Aarhus University, Gustav Wieds Vej 10D, Aarhus, Denmark; 2 Department of Biotechnology and Food Sciences, Norwegian University of Science and Technology, Sem Saelandsvei 6-8, Trondheim, 7491 Norway; 3 Genetics of Prokaryotes, Faculty of Biology and CeBiTec, Bielefeld University, Universitätsstraße 25, 33615 Bielefeld, Germany; 4 Institute of Molecular Microbiology and Biotechnology, University of Münster, Corrensstrasse 3, 48149 Münster, Germany

**Keywords:** *Bacillus methanolicus*, Thermophile, Methylotroph, Counter-selection system, Chromosomal deletion, Homologous recombination

## Abstract

**Background:**

*Bacillus methanolicus* is a promising candidate to become an industrial workhorse for methanol-based bioproduction due to its methylotrophy. However, genetic toolbox for this biotechnologically relevant thermophilic bacterium is still limited.

**Results:**

We here present the establishment of a counterselection system conducive to genome modifications in *Bacillus methanolicus* MGA3. We first identified four candidate genes or operons feasible to become counterselection markers: *lacZ* from *Bacillus coagulans*, *sacB* from *Bacillus subtilis*, *codBA* from *Escherichia coli*, and *oroP* from *Lactococcus lactis*, based on their absence from the genome of *B. methanolicus*. We tested substrates of the encoded enzymes to confirm their lack of toxicity to wild type *B. methanolicus.* Experimental results confirmed that none of the tested substrates affected the growth of *B. methanolicus* wild type at physiologically relevant concentrations. Subsequently, the selected genes were individually cloned into a low-copy plasmid pTH1mp and used to transform *B. methanolicus*. We evaluated the conversion of these non-toxic substrates to toxic products upon heterologous expression of the respective marker genes in *B. methanolicus*. The recombinant strains were demonstrated to possess the desired counterselection activity through lack of growth in the presence of their relevant substrate. A novel transconjugation method for high-efficiency plasmid-delivery of *B. methanolicus* was developed and used for the establishment of genome modification via non-replicating suicide vector designed for homologous recombination. Deletion of the chromosomal *upp* gene, crucial for uracil metabolism, was achieved using this method.

**Conclusions:**

In this study, we confirmed the utility of the established *oroP*-based counterselection system for the genome modifications through deletion of *upp* gene in *B. methanolicus*. The deletion strain exhibited reduced sensitivity to 5-fluorouracil, the toxic substrate of the *upp* encoded enzyme, demonstrating the practical application of the counterselection markers in genome engineering of *B. methanolicus*.

**Supplementary Information:**

The online version contains supplementary material available at 10.1186/s12934-025-02880-0.

## Background

*Bacillus methanolicus* is gaining increasing recognition as a production host using methanol as feedstock. Currently, most methanol is industrially produced from syngas, a gaseous mixture of hydrogen and carbon dioxide, commonly derived from natural gas [1]. However, novel technologies for methanol production that rely on renewable resources are being developed [2–6]. Moreover, the native ability of this bacterium to also catabolize mannitol as sole carbon and energy source has been utilized to demonstrate *B. methanolicus* growth on brown seaweed extracts [7].

Recently, the CRISPRi/dCas9 technique was developed and employed to repress gene expression in *B. methanolicus*, offering a valuable approach for characterization of gene function or redirection of carbon flux [8]. While CRISPRi/dCas9 enables to study essential genes by repressing their expression, it does not allow for permanent modifications of the genome and there is an urgent need to develop a technology that enables such modifications. Introducing changes directly into the genome presents several advantages over gene silencing, including plasmid-independent control of chromosomal gene expression levels through gene deletion or modification of regulatory elements upstream of the gene. Genome-based modifications are preferred for industrial strains due to their enhanced stability compared to plasmid-based strains and they are essential in developing platform strains with permanently integrated features, such as chassis organisms [9].

Marker-free gene deletion is a generally appraised method of genetic manipulation, with its major advantage of leaving no resistance marker gene in the genome [10]. It can be achieved using a non-replicating, suicide vector with selection and counterselection markers, which integrates into and is excised from the genome in a two-step process through the endogenous recombination systems of the host organisms. Counterselection markers are key tools in genome modification approaches which are typically composed of two steps. First, a suicide vector carrying two homology regions, and selection and counterselection markers is integrated into a genome via homologous recombination. The selection of the mutants with integrated plasmid occurs through cultivation in selective media supplemented with antibiotics. In a second recombination event, the integrated vector is excised from the integration site, yielding either the wild type or the expected mutant genotype. The selection is conducted using a counterselection marker on the plasmid which leads to the death of the cells harbouring it under defined growth conditions. Some of the counterselection markers commonly used in bacterial genome engineering are β*-*galactosidase encoded by *lacZ* or *bcl*, levansucrase encoded by *sacB*, cytosine permease and cytosine deaminase encoded by *codBA*, and orotate transporter encoded by *oroP*.

β-Galactosidase (encoded by *bgl* and *lacZ* genes; EC 3.1.1.26) catalyzes hydrolysis of the β−1,4 glycosidic bond of various glycoconjugates including 5-bromo-4-chloro-3-indolyl substrates such as 5-bromo-4-chloro-3-indolyl-β-d-galactopyranoside (X-gal) [11]. The product of X-gal hydrolysis is a toxic indoxyl precipitate. Derivatives of indoxyl were shown to inhibit the growth of a wide range of bacteria growing at various temperatures. For that reason, the β-galactosidase gene has the potential to become a widely used counterselection marker in a broad range of bacteria [11–13].

Levansucrase (sucrose:2,6-β-d-fructan 2,6-β-d-fructosyltransferase; EC 2.4.1.10) catalyses polymerization of sucrose into levan, a branched fructan polymer believed to have a structural or nutrient role for the cell [14–17]. The heterologous expression of levansucrase gene (*sacB*) from *B. subtilis* confers lethal sensitivity to sucrose in Gram-negative bacteria including thermophilic *Caldimonas thermodepolymeran*s but not in Gram-positive bacteria except of *Corynebacterium* sp [17–22]. The members of *Corynebacterium* genus, such as *C. glutamicum* and *C. diphtheriae* contain mycolic acid-rich outer membrane in the cell envelope, which may lead to retention of levansucrase and accumulation of levan in the periplasm similarly to Gram-negative bacteria [23–25]. The sucrose toxicity is likely caused by intracellular or extracellular accumulation of levan, which results in disruption of different cellular processes [17].

**Fig. 1:**
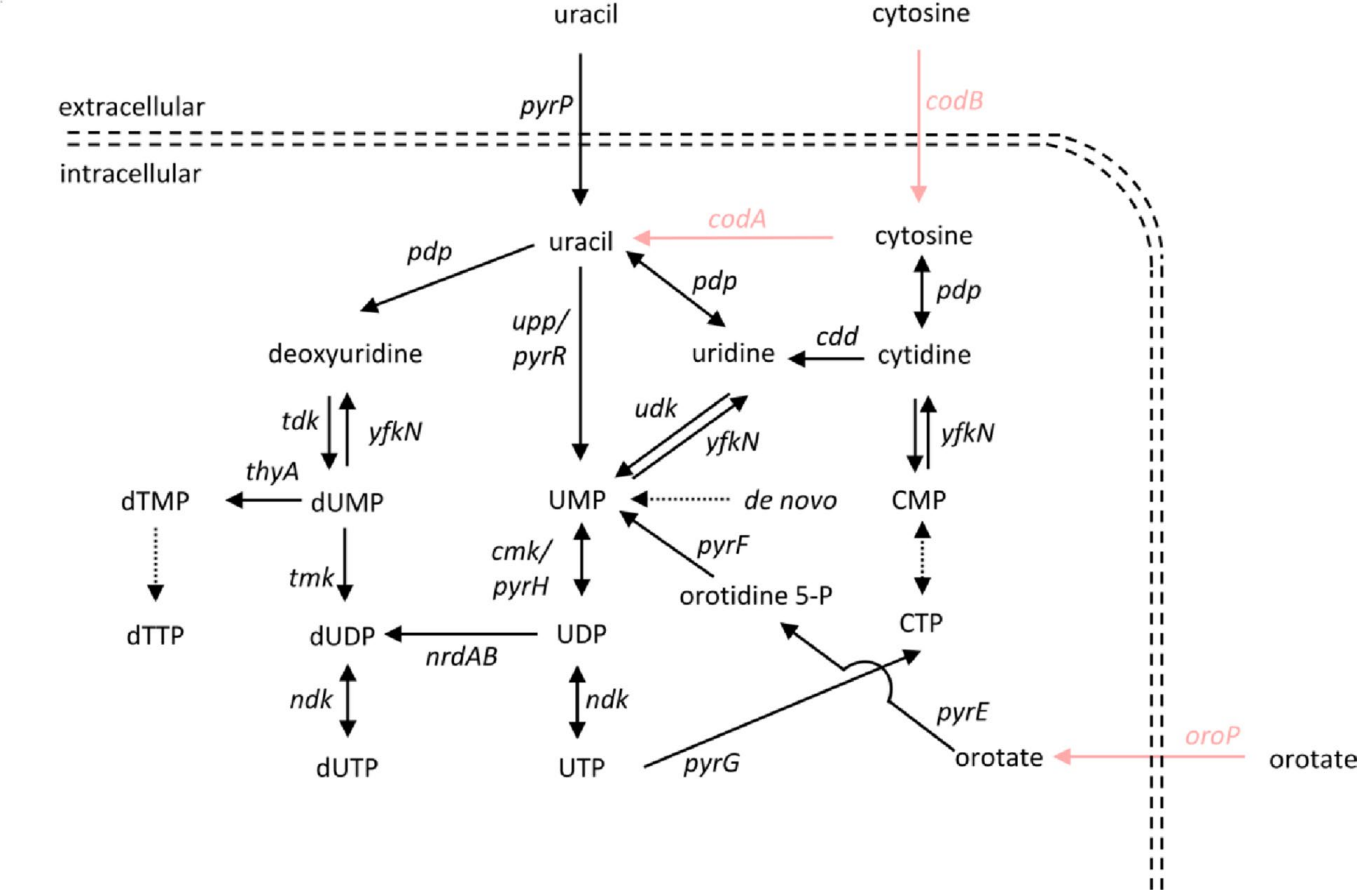
Simplified Kyoto Encyclopedia of Genes and Genomes (KEGG)-derived model of pyrimidine salvage pathways in *B. methanolicus*. Gene names of the respective enzymes are given. Arrows indicate the preferred direction of the catalysed reactions. Dotted arrows indicate multiple reactions. The genes in pink represent heterologous reactions explored here in counterselection marker testing. The gene symbols represent individual enzymes as follows: *cdd*, cytidine deaminase; *cmk*, cytidine 5′-monophosphate (CMP) kinase; *codA*, cytosine deaminase; *codB*, cytosine permease; *ndk*, nucleoside diphosphate kinase; *pdp*, pyrimidine-nucleoside phosphorylase; *pyrE*, orotate phosphoribosyltransferase; *pyrF*, orotidine 5′-monophosphate decarboxylase; *pyrG*, cytidine triphosphate (CTP) synthetase; *pyrH*, uridine 5′-monophosphate (UMP) kinase; *pyrP*, uracil permease; *pyrR*, pyrimidine operon attenuation protein/uracil phosphotransferase; *tdk*, thymidine kinase; *thyA*, thymidylate synthase; *tmk*, thymidine 5’-monophosphate (TMP) kinase; *udk*, uridine kinase; *upp*, uracil phosphoribosyltransferase; *yfkN*, 5’ nucleotidase; *oroP*, orotate transporter

Cytosine deaminase (CodA; EC 3.5.4.1) belongs to the pyrimidine salvage metabolism, and it catalyses the deamination of cytosine and its analogue, 5-fluorocytosine (5-FC), to uracil and toxic 5-fluorouracil (5-FU), respectively (Fig. 1) [26]. Genes encoding cytosine deaminase are less pervasive in bacterial genomes than *upp* encoding uridine phosphorylase, which also belongs to pyrimidine salvage pathway, and for that reason, often no previous gene deletions are required to establish a counterselection system [27]. Cytosine deaminase has been successfully used as a counterselection marker in *Bacillus licheniformis* and thermophilic *Thermus thermophilus* [26–28]. Additionally, it can be used in the base-editing technology, where it is fused with an engineered Cas9 protein to catalyse site-specific substitution of a cytidine with a thymine [29].

In some lactic acid bacteria, OroP is responsible for the uptake of orotate (TC 2.A.7.21.4), a precursor for pyrimidine biosynthesis present in milk and dairy products [30]. OroP confers 5-fluoroorotate (5-FO) sensitivity in *L. lactis* and, when heterologously overproduced, in *Escherichia coli* and *Bacillus subtilis* [31]. After 5-FO is imported into the cytoplasm by OroP, it is converted to 5-fluorouridine 5′-monophosphate (5-FUMP) in a two-step process by orotate phosphoribosyltransferase and orotidine 5′-monophosphate decarboxylase. 5-FUMP can be converted into 5-fluorodeoxyuridine 5′-monophosphate (5-FdUMP), a strong inhibitor of thymidylate synthase (ThyA) (Fig. 1) [32].

Just before the submission of this manuscript, a method for gene deletion in *B. methanolicus* was published. This method is restricted by some limitations such as long homology regions (2–6 kb) and the necessity to use an intermediate DNA host to prevent the activity of the restriction-modification system in *B. methanolicus* [33]. Here, we report testing of various counterselection markers in *B. methanolicus*, leading to the development and validation of a flexible and efficient counterselection system. Moreover, we present a plasmid delivery method using transformation via transconjugation to generate targeted genome modifications in *B. methanolicus* MGA3 using an *oroP*-containing suicide vector.

## Materials and methods

### Strains, plasmids, and primers

Bacterial strains, plasmids and primers (Sigma Aldrich) used in this study are listed in Tables 1 and 2. The *E. coli* strain DH5α was used as the general cloning host, *E. coli* MG1655 was used as a source of genomic DNA (gDNA) for cloning and *B. methanolicus* MGA3 was used as a host to test counterselection systems and the deletion method.

**Table 1:** Plasmids and bacterial strains used in this study

Name/abbreviated name	Relevant characteristics	Source
Plasmids		
pTH1mp	Cm^R^; derivative of pTH1mp-*lysC* for gene expression under control of the *mdh* promoter	[34]
pCasPP	Kan^R^; *P. polymyxa* genome editing vector, derivative of pUB110 (ATCC 37015)	[35]
pCS1966	Erm^R^, pCS1861-derivative carrying the *oroP* gene under control of synthetic promoter	[36]
pK19*mobsacB*	Kan^R^; empty mobilizable deletion vector for *C. glutamicum*, *lacZα*, *sacB*, *oriT*, *oriR*	[22]
pTH1xp-*lacZ*	Cm^R^; pTH1mcs-*lacZ* derivative for *lacZ* expression under control of the the xylose inducible promoter (P_*xyl*_)	[34]
pTH1mp-*oroP*^GTG^	Cm^R^; pTH1mp derivative for expression of *oroP* from pCS1966 under control of the *mdh* promoter	This work
pTH1mp-*lacZ*^GTG^	Cm^R^; pTH1mp derivative for expression of *lacZ* from pTH1xp-*lacZ* under control of the *mdh* promoter	This work
pTH1mp-*codBA*	Cm^R^; pTH1mp derivative for expression of *codBA* from gDNA of *E. coli* MG1655 under control of the *mdh* promoter	This work
pTH1mp-*sacB*	Cm^R^; pTH1mp derivative for expression of *sacB* from pK19*mobsac* under control of the *mdh* promoter	This work
pDELxo	Kan^R^, *B. methanolicus* genome editing vector, derivative of pCasPP, *oroP* counterselection marker under regulation of P_*xyl*_	This work
pDELxo*-Δupp*	Kan^R^, *B. methanolicus* suicide vector, derivative of pCasPP, *oroP* counterselection marker under regulation of P_*xyl*_; homology regions for upstream and downstream of the *upp* gene	This work
pTH1mp-*upp*	Cm^R^; pTH1mp derivative for expression of *upp* from *B. methanolicus* MGA3 under control of the *mdh* promoter	This work
Bacterial strains		
*E. coli* DH5α	General cloning host, F-*thi*−1 *endA*1 *hsdR*17(r-,m-) *supE*44 _l*acU*169 (_80*lacZ*_M15) *recA*1 *gyrA*96 *relA*1	Lab collection
*E. coli* MG1655	Wild type strain, used as *codBA* gene donor	Lab collection
*E. coli* S17-1	Strain used for transconjugation	DSMZ 9079
*B. methanolicus* MGA3	Wild type strain	ATCC 53907
*B. methanolicus* MGA3 Δ*upp*	MGA3 strain with deletion of *upp* gene	This work

Cm^R^, chloramphenicol resistant; Kan^R^, kanamycin resistant; Erm^R^, erythromycin resistant

**Table 2:** Primers used in this study

Primer name	Sequence 5′→3′	Target
C025	*TACATAAATAGGAGGTAGTACAT*GTGTCGCAAGATAACAACTTTAGCCAG	*codB*^*GTG*^*A*; fw
C026	*CCGGGAATTCAAGCTTTAAA*TCAACGTTTGTAATCGATGGCTTCTG	*codB*^*GTG*^*A*; rv
C035	*TACATAAATAGGAGGTAGTACAT*ATGAACATCAAAAAGTTTGCAAAACAAGCA	*sacB*; fw
C036	*CCGGGAATTCAAGCTTTAAA*TTATTTGTTAACTGTTAATTGTCCTTGTTCAAGG	*sacB*; rv
CS02	*TAAACAATTACATAAATAGGAGGTAGTACAT*GTGTATATTTACCTAGCTTTTGCATTAGTT	*oroP*^*GTG*^; fw
CS03	*TAGACCTATGGCGGGTACCATATG*TTATAAAAATTTAATGAATATTATTCCGGTCAG	*oroP*; rv,
CS07	*TAAACAATTACATAAATAGGAGGTAGTACAT*GTGCTAAAAAAACATGAAAAATTTTATTACG	*lacZ*^*GTG*^; fw
CS08	*TAGACCTATGGCGGGTACCATATG*CTATTTTTCAATTACCTGCAAAATTTTCAC	*lacZ*, rv
C028	GCCCATAACTGAAAACAGCCTGG	*codA*; seq1; fw
C029	GATGTTTGCCATTCCGGTGG	*codB*; seq1; fw
C030	CACTGTGATGAGATCGATGACGAG	*codA*; seq2; fw
C031	CGATAACGCACTCTATGCGTCG	*codB*; seq2; fw
C037	CACAAGAATGGTCAGGTTCAGCC	*sacB*; seq; fw
VPJF	TCTAATCCTTCTAAAAAATATAATTTAGAAAACTAAG	pTH1mp; seq; fw
VPJR	GGTGCGGGCCTCTTCGCTATTACG	pTH1mp, seq; rv
DEL01	CAGCTCGCGGACGTGCTCATAGT	pCasPP backbone; fw
DEL02	TTTAAGGGTTTTCAATACTTTAAAACACATACA	pCasPP backbone; rw
DEL09	*TAAAGTATTGAAAACCCTTAAA*GGGACGTCGACTCTAACTTAT	P_*xyl*_; fw
DEL10	CCATTTCCCCCTTAAGTGAAC	P_*xyl*_; rv
DEL11	*TTCACTTAAGGGGGAAATGGCAA*GTGTATATTTACCTAGCTTTTGCATTAGTT	*oroP*^GTG^; fw
DEL12	*ATGAGCACGTCCGCGAGCTG*CAAGCTCTAGACCTATGG	*oroP*^GTG^; rv
DEL25	*ACCGGCGCATCAAGCCCGCCGA*TCGACAGGAATAATTCTTTGAAAGAT	*upp*; LF; fw
DEL26	TTTGGAACGAAATAAGGAAAAAGAAG	*upp*; LF; rv
DEL27	*CTTTTTCCTTATTTCGTTCCAAA*ATAAACTTTTGCCATGGCAATC	*upp*; RF; fw
DEL28	*CTTTTTACGGTTCCTGGCCA*CACGAGAACATAAGCCTAAATTGA	*upp*; RF; rv
DEL29	CTCAGGAAAATGTGGGGTATG	*upp*; confirmation; fw
DEL30	TCAACTTCAGCGGAGTTCA	*upp*; confirmation; rv
DEL57	*TAAACAATTACATAAATAGGAGGTAGTACAT*ATGGCAAAAGTTTATGTTTTTGATCATCCAC	*upp*; fw
DEL58	*TAGACCTATGGCGGGTACCATATG*TTATTTCGTTCCAAATAATCGGTCTCCT	*upp*; rv

Italic sequences represent homology regions for Gibson assembly reaction

### Molecular cloning

The *E. coli* DH5α competent cells were prepared according to the calcium chloride protocol as described in [37]. All standard molecular cloning procedures were carried out according to manuals provided by manufacturers. Chromosomal DNA was isolated as described in [38]. PCR products were amplified using CloneAmp HiFi PCR Premix (Takara) and purified using a QIAquick PCR Purification Kit from Qiagen. DNA fragments were separated using 8 g L^− 1^ SeaKem LE agarose gels (Lonza) and isolated using a QIAquick Gel Extraction Kit (Qiagen). Recombinant DNA was assembled in vitro by means of the isothermal DNA assembly method [39]. Colony PCR was performed using GoTaq DNA Polymerase (Promega). The sequences of cloned DNA fragments were confirmed by Sanger sequencing (Eurofins). For initial testing of counterselection systems, *B. methanolicus* MGA3 cells were made electrocompetent and transformed by electroporation as described previously [40].

### Growth media and conditions for shake flask cultivations

*E. coli* strains were cultivated at 37 °C in Lysogeny Broth (LB) or on LB agar plates supplemented, when necessary, with antibiotics: chloramphenicol at 15 µg mL^−1^, kanamycin at 50 µg mL^−1^ or erythromycin at 150 µg mL^−1^. For standard cultivations, recombinant strains of *B. methanolicus* were cultivated in MVcM minimal medium with 200 mM methanol supplemented with 5 µg mL^−1^ chloramphenicol when necessary [41]. For precultures, minimal medium supplemented with 0.25 g L^−1^ yeast extract, designated MVcMY, was used. Unless specified otherwise, cultivations were performed in triplicate in 250 mL baffled flasks (40 mL, 200 rpm, 50 °C), inoculated to a starting optical density at 600 nm (OD_600_) = 0.2. Growth was monitored by measuring OD_600_ with a cell density meter (WPA CO 8000 Biowave). Specific growth rates were calculated from the exponential phase, by calculating the slope of semi-logarithmic plots of OD_600_ versus time. The effect of 5-FO on *B. methanolicus* MGA3 (pTH1mp) and *B. methanolicus* MGA3 (pTH1mp-*oroP*), and X-gal on *B. methanolicus* MGA3 (pTH1mp) and *B. methanolicus* MAG3 (pTH1mp-*lacZ*) was evaluated upon cultivation in 250 mL shake flasks.

For the evaluation of MGA3 (pTH1mp-*codBA*) and MGA3 (pTH1mp-*sacB*) strains, the growth experiments were performed in 24-well microtiter plates (Duetz system, Adolf Kühner AG) in 3 mL MVcMY medium at 50 °C and 200 rpm. The *B. methanolicus* cultures were inoculated from an overnight culture to an OD_600_ between 0.1 and 0.2 in triplicate and the OD_600_ was measured every 2 h. The MGA3 (pTH1mp-*codBA*) strain was cultivated with the antimetabolite 5-FC and MGA3 (pTH1mp-*sacB*) with sucrose. The effect of supplementation with sucrose, 5-FC and 5-FU was also evaluated in 24-well microtiter plates (Duetz system, Adolf Kühner AG).

### Preparation of crude extracts and enzyme assay for β-galactosidase

For evaluation of β-galactosidase activity, overnight cultures of *B. methanolicus* strains MGA3 (pTH1mp-*lacZ*) and MGA3 (pTH1mp) were diluted to OD_600_ 0.2 in fresh MVcMY medium with appropriate antibiotics and cultivated until OD_600_ 1–1.5. 30 mL of cells were harvested by centrifugation (5,000 x *g*, 5 min, 4 °C), and the pellets were stored at −80 °C. Cells were thawed, resuspended in 2 mL Tris buffer (100 mM, pH 7.2) and sonicated on ice/water using Fisherbrand Sonic Dismembrator (FB-505) with 40% amplitude with 2 s on and 1 s off pulse cycles for 6 min. Cell debris was then removed by centrifugation (21,000 x *g*, 1 h, 4 °C). Protein concentrations were determined by Bradford assay using bovine serum albumin (Sigma) as a standard [42].

Enzymatic activities were measured in the crude extract by monitoring the liberation of *o*-nitrophenol from *o*-nitrophenyl β-d-galactopyranoside (ONPG) at 420 nm. Z-buffer pH 7.2 (900 µL), 20 mg mL^− 1^ ONPG (50 µL) and 1 M dithiothreitol (DTT) (1 µL) were mixed, and the catalysis started by the addition of cell extract (50 µL). Measurements were executed at 50 °C. The molar extinction coefficient for *o*-nitrophenyl at 420 nm and pH 7.2 used for calculation is 4500 M^− 1^ cm^− 1^ and the light path 1 cm. One unit (U) is defined as the amount of enzyme able to convert 1.0 µmol of ONPG per min [43].

###  Transformation of *B. methanolicus* via transconjugation with *E. coli* S17-1

*B. methanolicus* MGA3 was subjected to transconjugation by using a donor strain *E. coli* S17-1 harbouring the suicide plasmid with specific homologous regions. The process of transconjugation and homologous recombination between the introduced suicide vector and the genome was based on [35]. Here, a modified procedure is presented. 25 mL SOB medium was inoculated with *B. methanolicus*, and 25 mL LB medium supplemented with 25 µg mL^− 1^ kanamycin was inoculated with *E. coli* S17-1. The cultures were incubated for 16 h at 200 rpm and 50 °C and 37 °C for *B. methanolicus* and *E. coli* S17-1, respectively. Pre-cultures were sub-cultured 1:100 into 50 mL of fresh media and incubated for 4 h until the early exponential growth phase. After cultivation, 9 mL or 4.5 mL of the recipient strain culture was mixed with 3 mL or 1.5 mL of the donor strain culture, respectively. Cells were then harvested by centrifugation (8,000 *x* *g*, 3 min), the supernatant was discarded, and the cells were dropped on non-selective SOB plates and incubated overnight at 40 °C. The next day, cells from each plate were collected using sterile pipette tips, and they were resuspended in 500 µL of 0.9% NaCl before being plated on selective SOB plates with 25 µg mL^− 1^ kanamycin. In this step, the clones were selected where the first recombination event had occurred. Plates were then incubated (50 °C, 24–72 h) until bacterial colonies appeared. In the next step, colonies were picked and transferred to SOB plates supplemented with 80 µg mL^− 1^ 5-FO and xylose (10 g L^− 1^) to select for clones where the second recombination step and plasmid curing had occurred. Colonies were picked for colony PCR using specific primers for verification of deletion (DEL29 and DEL30).

### Genome sequencing and variant calling

gDNA was extracted using the Monarch Genomic DNA Purification Kit (New England Biolabs) following the supplier’s protocol. The whole genome sequencing was outsourced to Eurofins Genomics. Two paired-end libraries, with average insert sizes of approximately 500 bp and 2,000 bp, were prepared for sequencing on the Illumina GA IIx platform (Illumina Inc., San Diego, CA, USA). The resulting data underwent quality control to eliminate reads containing more than five ambiguous bases, reads with at least 20 bases below Q20 quality, adapter sequences, and duplicates. After filtering, high-quality paired-end reads were retained, achieving a sequencing depth of ~ 100×.

Trimmed sequencing reads were aligned against the complete reference genome of *Bacillus methanolicus*, including its chromosome and the two native plasmids, pBM19 and pBM69 (GenBank accessions: CP007739, CP007740, CP007741) as described by Hilker et al. [44]. Read preprocessing involved quality trimming using Trimmomatic v0.33, retaining sequences longer than 36 bp [45]. High-quality reads were mapped using Bowtie 2 [46]. Read alignment outputs were examined and curated using ReadXplorer v2.2.2 [44]. For variant detection, the Bcftools mpileup pipeline was applied, with the quality cut-off scores set to ≥ 10 for total read depth (DP) and ≥ 15 mean mapping quality (MQ) [47].

## Results

### Testing and evaluation of potential counterselection markers for *B. methanolicus*

To establish a counterselection system for *B. methanolicus* we have selected and assessed potential candidates based on the following criteria: (1) the absence of the candidate genes in the genome of *B. methanolicus*, (2) the lack of toxicity of the counterselection substrates to *B. methanolicus* wild type and (3) the ability of the selected counterselection enzymes to catalyse the reaction wherein the product from a non-toxic substrate is toxic to *B. methanolicus*. We considered the following counterselection markers: *B. subtilis*-derived levansucrase encoded by *sacB*, *lacZ* gene derived from *B. coagulans* DSM1 encoding β*-*galactosidase, *E. coli*-derived *codBA* encoding cytosine permease and cytosine deaminase, and *oroP* coding for orotate transporter from *L. lactis.* A BLASTP query of the *B. methanolicus* genome confirmed that no potential homologues to any of these candidate genes were found (data not shown).

We then tested if the substrates of the chosen counterselection markers are toxic to the wild-type *B. methanolicus* and to the empty vector control strain MGA3(pTH1mp), depending on the specific experimental setup. The following compounds were tested: (1) sucrose whereof hydrolysis into glucose and fructose polymer is catalysed through the action of the enzyme levansucrase encoded by *sacB*; (2) X-gal which undergoes hydrolysis through catalytic activity of β-galactosidase encoded by *lacZ* wherein a toxic indoxyl precipitate is created; (3) 5-FC which is converted to toxic 5-FU in the process of deamination catalysed by cytosine deaminase encoded by *codBA* (Fig. 1); (4) 5-FO which is imported into the cell by OroP transporter encoded by *oroP* and subsequently converted to toxic 5-FdUMP [11, 26–28, 32]. As presented in Table 3, the tested compounds hardly affected the growth of *B. methanolicus*, confirming that none of them is toxic to this organism.

**Table 3:** Effect of selected counterselection marker substrates on growth of *B. methanolicus* MGA3 and empty vector control strain MGA3(pTH1mp)

Strain	µ (h^− 1^) in the presence of different additives				
	No additive	Sucrose (10%)	X-Gal (20 µg mL^− 1^)	5-FO (100 µg mL^− 1^)	5-FC (100 µg mL^− 1^)
MGA3	0.46 ± 0.04	0.48 ± 0.01	n.t.	n.t.	0.42 ± 0.01
MGA3 (pTH1mp)	0.47 ± 0.02	0.44 ± 0.01	0.46	0.44 ± 0.01	n.t.

The means of replicated cultivations (*n* = 2–6) are shown. Standard deviations are shown for *n* ≥ 3. n.t. – not tested

To test the activity of selected counterselection markers in *B. methanolicus*, we cloned the respective genes into the low-copy-number rolling circle plasmid pTH1mp under the control of the methanol dehydrogenase (*mdh*) promoter. This led to the creation of expression plasmids pTH1mp-*sacB*, pTH1mp-*lacZ*, pTH1mp-*codBA* and pTH1mp-*oroP* which together with a control empty vector, pTH1mp, were used to transform *B. methanolicus* MGA3 and generate the following strains: MGA3 (pTH1mp-*sacB*), MGA3 (pTH1mp-*lacZ*), MGA3 (pTH1mp-*codBA*), MGA3 (pTH1mp-*oroP*) and MGA3 (pTH1mp). The functional expression of heterologous β-galactosidase LacZ was confirmed by enzyme assays in crude extracts, revealing LacZ activity in *B. methanolicus* MGA3 (pTH1mp-*lacZ*) of 10.1 ± 1.1 U mg^− 1^ protein in comparison to < 0.2 U mg^− 1^ protein in the control strain *B. methanolicus* MGA3 (pTH1mp).

**Fig. 2:**
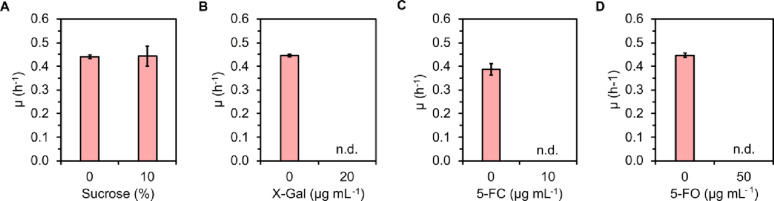
Effect of the counterselection markers on the growth of *B. methanolicus* recombinant strains in the presence of their respective substrates. The following strains were tested: **A** MGA3 (pTH1mp-*sacB*), **B** MGA3 (pTH1mp-*lacZ*), **C** MGA3 (pTH1mp-*codBA)*, **D** MGA3 (pTH1mp-*oroP*). The means of triplicates with standard deviations are shown. n.d. – not detected

The recombinant *B. methanolicus* strains were then cultivated with the respective substrates for counterselection markers at various concentrations. No growth inhibition was observed upon addition of sucrose to the growth medium of levansucrase-expressing strain, MGA3 (pTH1mp-*sacB*) (Fig. 2A). While supplementation with X-gal at 20 µg mL^− 1^ did not affect the empty vector control strain, MGA3 (pTH1mp) (Table 3), this resulted in inhibited growth of MGA3 (pTH1mp-*lacZ*) heterologously overproducing β*-*galactosidase (Fig. 2B). The *codBA* expression strain, MGA3 (pTH1mp-*codBA*), did not grow when 10 µg mL^− 1^ 5-FC was supplemented to the cultivation medium (Fig. 2C), while the wild-type MGA3 strain grew well in the presence of up to 100 µg mL^− 1^ 5-FC. Similarly, MGA3 (pTH1mp) grew well upon supplementation of 100 µg mL^− 1^ 5-FO, however, supplementation of 50 µg mL^− 1^ 5-FO to the growth medium of MGA3 (pTH1mp-*oroP*) resulted in complete growth arrest (Fig. 2D).

Based on these findings, it was concluded that *lacZ*, *codBA* and *oroP* could serve as counterselection markers in *B. methanolicus*. Among these, we chose to construct the deletion vector using *oroP* as a counterselection marker, based on its small molecular size of 0.9 kb in comparison to 2.5 kb and 2 kb for *codBA* and *lacZ*, respectively.

### Development of a transconjugation method for plasmid delivery to *B. methanolicus*

The rates of homologous recombination vary across bacterial species; however, it can be assumed that they are relatively constant within one species [48]. Considering this fact, to develop homologous recombination (HR)-based method for genome modification, the foreign DNA delivery method needs to be optimised. Until now, *B. methanolicus* has been transformed with plasmid DNA through chemical treatment of protoplasts or electroporation [33, 40, 49]. Here, we decided to follow another approach, namely transconjugation, which is often a preferred method when coupled to homologous recombination since plasmid DNA (pDNA) enters the recipient cell as single-stranded DNA. To do so, we used the previously developed plasmid pCasPP carrying *oriT* necessary for the transformation via interspecies transconjugation with the *E. coli* S17-1 donor strain carrying chromosomally integrated conjugal transfer machinery [35]. The protocol was adapted by changing the temperature of co-cultivation to that suitable for both *B. methanolicus* and *E. coli*. After a preliminary test with the growth of both organisms on agar plates at different temperatures, we decided to use 40 °C, which supported the growth of both species. The optimised protocol was successfully used for the plasmid delivery of *B. methanolicus* with an efficiency of around 600 colonies/conjugation in the preliminary experiment, as opposed to ~ 20 colonies µg^− 1^ pDNA obtained in the classic electroporation protocol.

### Construction of a suicide vector for genome modification in *B. methanolicus*

**Fig. 3:**
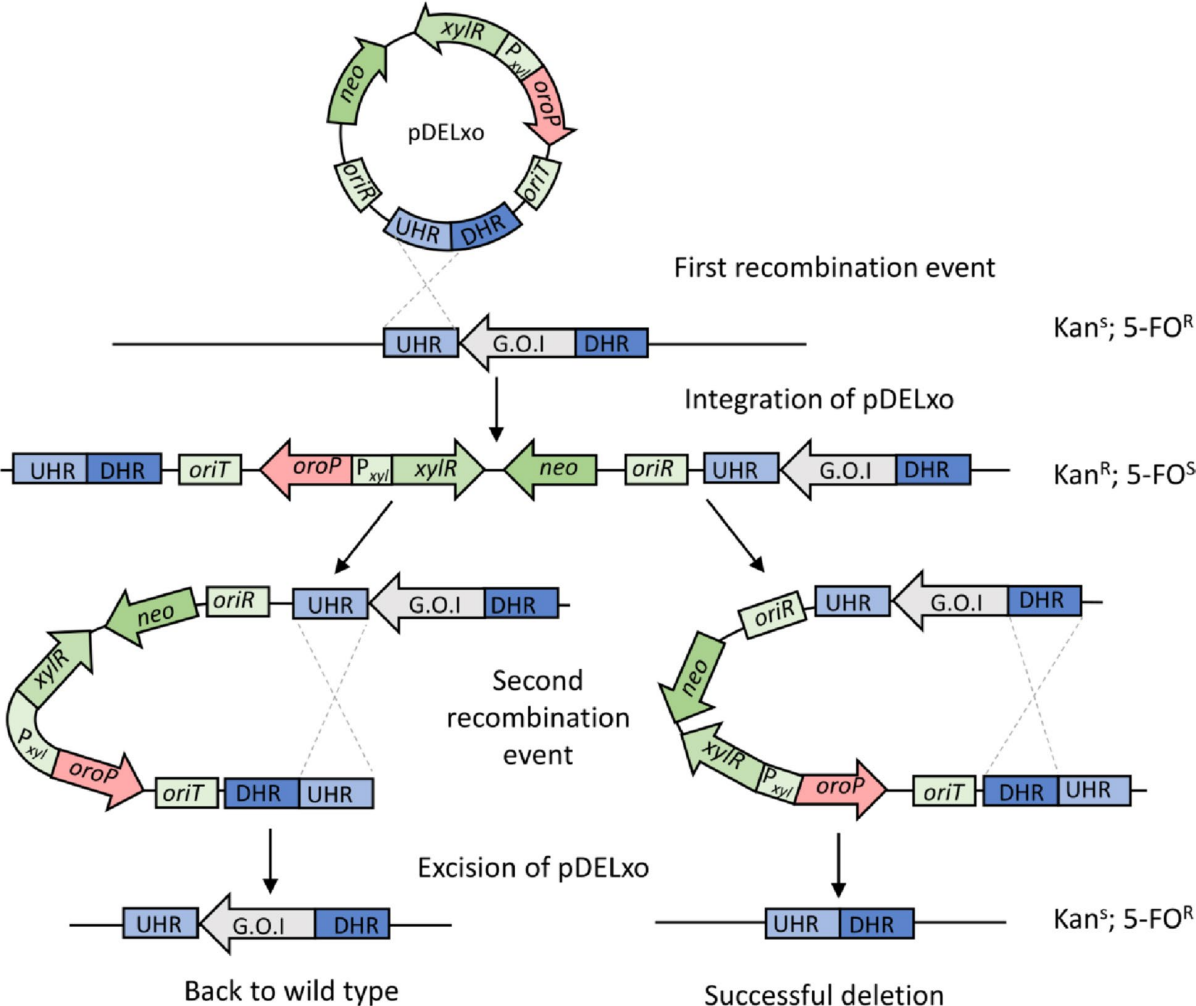
HR-mediated genome modification method for *B. methanolicus* MGA3. UHR, upstream homology region; DHR, downstream homology region; G.O.I., gene of interest; P_*xyl*_, xylose inducible promoter; *xylR*, transcriptional regulator gene; *oroP*, orotate transporter encoding gene; *oriR*, origin of replication; *oriT*, origin of transfer; *neo*, kanamycin and neomycin resistance gene; Kan^R^, kanamycin resistant; 5-FO^R^, 5-FO resistant; Kan^S^, susceptible to kanamycin; 5-FO^S^, susceptible to 5-FO

We next decided to use the transconjugation method for plasmid delivery to enable genomic modification in *B. methanolicus* via HR. To do so, we designed a respective vector which should meet several requirements: (1) plasmid delivery via transconjugation; (2) genome modification through a double homologous recombination event; (3) testing of the first recombination event with antibiotic resistance; (4) testing of the second recombination event with a counterselection marker. To meet these prerequisites, the vector should possess *oriR* for replication in *E. coli*, *oriT* for transformation via transconjugation, singular restriction for cloning of homologous regions, and selection and counterselection markers, and no *repU* gene encoding pUB110 replication initiation protein for *B. methanolicus* [34, 50]. Lack of *repU* gene will ensure that cells which propagate on the selection plate undergo one HR event wherein the suicide vector is inserted into the genome. The presence of the counterselection marker is necessary to test for the cells where the second HR event occurred, which means removal of the suicide vector from the genome leading to creation of the strain with the desired modification in the genome or restoration of the wild type strain (Fig. 3).

The suicide vector was constructed based on the pCasPP plasmid used to establish the transconjugation method. To do so, the pCasPP backbone fragment, containing the kanamycin resistance gene, the *oriR*, *oriT* and *Spe*I restriction site for cloning of the homology regions, the xylose-inducible promoter together with the regulator-encoding gene from pBV2xp vector, and *oroP* gene from pCS1966 were amplified via PCR. All elements were combined via Gibson Assembly to render pDELxo deletion vector that was used to introduce genome modification in *B. methanolicus* (Fig. 3). Next, the newly designed and constructed vector was tested, and we have selected the *upp* gene encoding uracil phosphoribosyltransferase as a first target.

### Successful application of the newly established method for the chromosomal deletion of the *upp* gene in *B. methanolicus*

In *B. methanolicus*, uracil is converted to uridine 5′-monophosphate (UMP), a precursor of pyrimidine nucleotides, in the reaction catalysed by pyrimidine salvage enzyme uracil phosphoribosyltransferase encoded by *upp* (BMMGA3_16035) (Fig. 1). We chose the *upp* gene as a first deletion target, based on the genome-based model for uracil metabolism in *B. methanolicus*, predicting that its deletion should affect the physiology of *B. methanolicus* under supplementation with 5-fluorouracil.

Since the predicted uracil salvage pathway of *B. methanolicus* resembles that of *B. subtilis*, we expected that deletion of *upp* should not be lethal to *B. methanolicus*, as UMP can be synthesised not only from uracil but also in the *de novo* biosynthetic pathway (Fig. 1). While uracil serves as a precursor for pyrimidine biosynthesis, the supplementation of growth media with 5-FU leads to the formation of 5-FUMP by uracil phosphoribosyltransferase. 5FUMP is converted to 5-FdUMP in reactions as shown in Fig. 1. 5-FdUMP inhibits the activity of thymidylate synthase, which methylates deoxyuridine 5′-monophosphate (dUMP) to deoxythymidine 5′-monophosphate (dTMP), resulting in decreased DNA synthesis, repair, and lastly cell death (Fig. 1). This means that the growth of wild-type *B. methanolicus* is likely inhibited by the supplementation with 5-FU. To test that hypothesis, we have cultivated *B. methanolicus* in the presence of increasing concentrations of 5-FU. Supplementation with 0.65 µg mL^− 1^ of this compound led to complete growth arrest, confirming that 5-FU is toxic to wild-type *B. methanolicus* (Table 4).

**Table 4:** Effect of 5-FU on growth rates and biomass formation of *B. methanolicus* MGA3 (pTH1mp), MGA3 Δ*upp* (pTH1mp) and a complementation strain MGA3 Δ*upp* (pTH1mp-*upp*)

Strain	5-FU (µg mL^− 1^)	µ (h^− 1^)	Max. ΔOD_600_
MGA3 (pTH1mp)	0.00	0.35 ± 0.01	7.87 ± 0.50
	0.65	n.d.	< 0.5
MGA3 Δ*upp* (pTH1mp)	0.00	0.39 ± 0.01	7.60 ± 0.28
	0.65	0.20 ± 0.01	4.67 ± 1.31
MGA3 Δ*upp* (pTH1mp-*upp*)	0.00	0.35 ± 0.00	8.07 ± 0.41
	0.65	0.10 ± 0.01	3.00 ± 0.71

The means of replicates (*n* = 3) with standard deviations are shown. n.d. – not detected

**Fig. 4:**
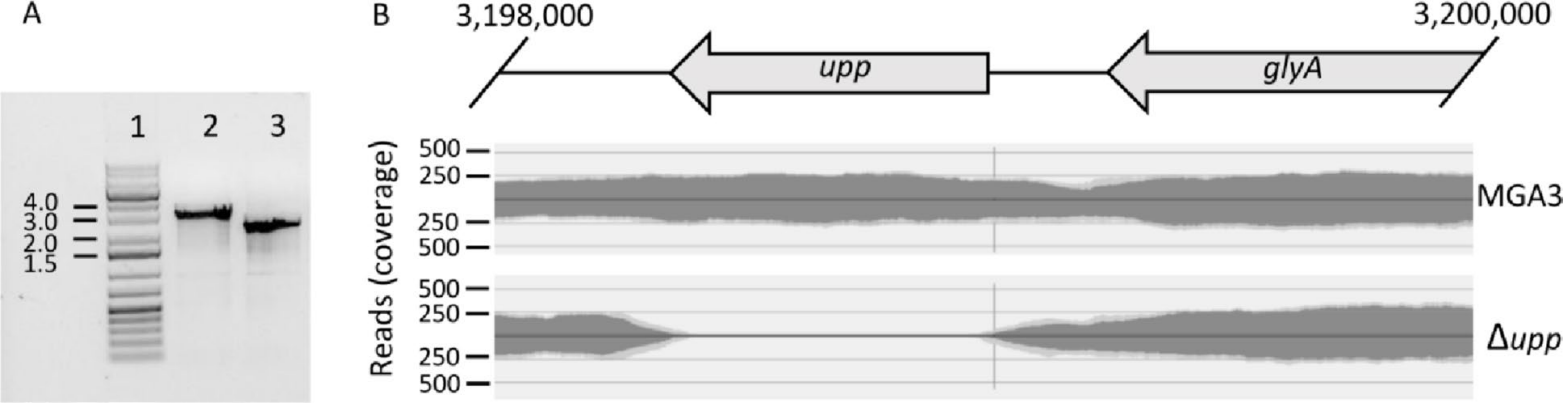
Confirmation of chromosomal *upp* gene deletion in *B. methanolicus*. **A** Agarose gel electrophoresis picture of PCR products obtained with primers DEL29 and DEL30 using chromosomal DNA of wild type strain (lane 2), and deletion strain (lane 3). Expected product sizes are 2813 bp and 2213 for lanes 2 and 3, respectively. Lane 1 shows the DNA ladder. **B** Alignment of genome sequencing reads for MGA3 and the Δ*upp* strains

The chromosomal *upp* gene was deleted in *B. methanolicus* using the method established in this study, i.e. transconjugation with a suicide vector which possesses 1 kb DNA fragments homologous to genome regions upstream and downstream of *upp* gene. After the transconjugation, total of 16 colonies was obtained on the kanamycin selection plate and transferred to the counterselection plate supplemented with 80 µg mL^− 1^ 5-FO and 10 g L^− 1^ xylose. All colonies survived on the counterselection plate, and they underwent colony PCR after the overnight incubation at 50 °C. Out of 16 colonies, all showed a band size characteristic for a deletion mutant (Supplementary Figure S1A), and one was chosen for the detailed analysis (Fig. 4A). The deletion was confirmed via DNA agarose gel electrophoresis of PCR products, Sanger sequencing and whole-genome sequencing (Fig. 4). Together with the genome of MGA3 Δ*upp* strain, the genome of the wild type MGA3 strain was re-sequenced, and sequencing reads of both genomes were aligned against the complete reference genome of *B. methanolicus* published 2014 [51]. These alignments were used for detection of single nucleotide polymorphisms (SNP) present in the newly sequenced genomes. Total 43 SNP were detected in the genome of *B. methanolicus* MGA3, and 39 in the genome of MGA3 Δ*upp*, with 32 SNPs shared in both genomes (Supplementary Figure S2). The 7 unique SNPs in the genome of MGA3 Δ*upp* are either single nucleotide changes within coding sequences of two hypothetical proteins or in non-coding region of the genome (Supplementary Table S1), suggesting no off-target changes in the genome. To exclude polar effect caused by the deletion, we inspected the genomic organisation of the *upp* gene. The *upp* gene is located 456 bp upstream of BMMGA3_16030 encoding a nuclease-related domain (NERD)-containing protein, and 4 putative promoter sequences are detected with SAPPHIRE tool in that region but none inside the *upp *sequence with *P* < 0.01 indicating that regulatory regions were not disrupted when *upp *was deleted [52]. However, no TSS was assigned to BMMGA3_16030 in a genome-scale transcriptomic analysis, so this conclusion is not confirmed experimentally [53]. Additionally, we performed the deletion complementation through plasmid-based overexpression of the *upp* gene. The wild-type phenotype was not fully restored, as the MGA3 Δ*upp* (pTH1mp-*upp*) could grow in the presence of the 5-FU, albeit at lower growth rate and to lower final OD_600_ in comparison to MGA3 Δ*upp* (pTH1mp) (Table 4).

The physiological effect of the deletion of *upp* gene was evaluated through the cultivation of *B. methanolicus* MGA3 Δ*upp* (pTH1mp) strain in MVcM minimal medium supplemented with the toxic uracil analogue 5-FUunder the assumption that the deletion of *upp* would decrease the toxic effect of 5-FU on *B. methanolicus*. The deletion of *upp* gene did not have a major effect on growth rate and biomass formation under control conditions without supplementation with 5-FU (Table 4) which aligns with the SNP analysis (Supplementary Figure S2 and Table S1) where no significant differences to the wild-type genome were found. However, contrary to the wild type *B. methanolicus*, the supplementation of cultivation media with 5-FU at 0.65 µg mL^− 1^ did not lead to growth arrest of *B. methanolicus* MGA3 Δ*upp* (pTH1mp), but rather to the decrease of its growth rate and biomass formation. This demonstrated that *upp* gene product is in fact involved in uracil metabolism in *B. methanolicus* and seems to play a role in pyrimidine biosynthesis.

## Discussion

In this work, we tested the conditional toxic effect of the expression of gens for either *lacZ*-encoded β-galactosidase, *sacB*-encoded levansucrase, *codBA*-encoded cytosine permease with cytosine deaminase and orotate transporter encoded by *oroP* on the thermophilic methylotroph *B. methanolicus*. Based on the preliminary results, we selected and evaluated the use of *oroP* as a potential counterselection marker and showed its applicability for the generation of markerless modifications in the genome of *B. methanolicus.* This was achieved by using a new transconjugation method developed here and through the integration of a suicide vector with 1-kb homology regions and the implementation of a combination of 5-FO transport and conversion to toxic 5-FdUMP for counterselection of the second recombination event.

A strong inhibitory growth effect was observed upon expression of three out of four tested candidate genes in combination with their respective substrates, while *sacB* gave no toxic effect in the presence of sucrose in concentrations up to 10%. This could have been due to the fact that levan does not seem to be toxic to Gram-positive bacteria with the exception of *Corynebacterium* sp. and *Streptococcus* sp [17–21]. The toxicity of levan is likely caused by its retention in the cell envelope of some Gram-positive bacteria similar to the accumulation of levan in the periplasm of Gram-negative bacteria [23–25]. Considering the similarity of the cell wall between *Bacillus* species, the lack of levan toxicity on *B. methanolicus* is conceivable. However, since the functional expression of *sacB* has not been confirmed experimentally, it cannot be excluded that no levan toxicity could have been caused by low expression or enzymatic activity leading to insufficient intracellular levan accumulation.

The growth inhibition in the *lacZ*-expressing strains stems from the intracellular cleavage bromo-4-chloro-3-indolyl substrates such as X-gal and accumulation of toxic indoxyl precipitates. Sensitivity to products of cleavage of bromo-4-chloro-3-indolyl compounds was observed for other Gram-negative and Gram-positive bacteria, including those belonging to the genera *Bacillus*, *Corynebacterium*, *Micrococcus*, *Paracoccus*,* Rhodococcus*, *Staphylococcus*, *Thermus* and *Xanthomonas*. The cleavage of bromo-4-chloro-3-indolyl compounds by *lacZ*-encoded β-galactosidase has the potential to be used for selection purposes in a broad range of microorganisms, including thermotolerant organisms such as *B. smithii*, *T. thermophilus*, and *B. methanolicus* [11, 12].

The operon *codBA* and gene *oroP* are involved in the pyrimidine metabolism, wherein *codB* and *oroP* encode transporters of cytosine and orotate, respectively, and *codA* for cytosine deaminase. Cytosine deaminase catalyses the conversion of cytosine to uracil, while orotate can be transformed to UMP by native metabolism of *B. methanolicus*. In both cases, when fluorinated derivatives are imported into the cell through their transporters, they are converted either by heterologous or native enzymes to toxic metabolites. Neither 5-FC nor 5-FO are toxic to *B. methanolicus* in concentrations up to 100 µg mL^−1^, however, as low as 10 µg mL^−1^ of 5-FC completely arrests the growth of *codBA* expressing *B. methanolicus* strain and 50 µg mL^−1^ 5-FO arrests the growth of *oroP* expressing strain. Similarly, the growth of various *Bacillus* species, such as *B. licheniformis*, *B. subtilis*, *B. megaterium*, *B. amyloliquefaciens* is not inhibited by 5-FC at a concentration of up to 1000 µg mL^−1^ while 2 µg mL^−1^ arrests the growth of *codBA* expressing *B. licheniformis* and 20 µg mL^− 1^ 5-FO arrests the growth of *oroP* expressing *B. subtilis* [27, 54].

*B. methanolicus*, like *B. subtilis*, possesses two genes, *upp* and *pyrR*, encoding uracil phosphoribosyltransferase, which catalyses the conversion of uracil to UMP [55]. When 5-FU is used as a substrate for this enzyme, it leads to the accumulation of toxic metabolite 5-FdUMP an inhibitor of thymidylate synthase. Very low concentrations of 5-FU affect the growth of *B. methanolicus*, with 0.65 µg mL^−1^ 5-FU fully arresting its growth. This is in a similar range to other *Bacillus* species, whose growth is inhibited by 5-FU concentrations lower than 4 µg mL^−1^, for example, in the case of *B. subtilis*, growth inhibition occurred at 1.3 µg mL^−1^ 5-FU and a complete growth arrest at 3.9 µg mL^−1^ [27, 56]. The 5-FU toxicity threshold differs quite drastically between bacterial genera, wherein growth arrest for wild-type *Pseudomonas protegens* by 5-FU supplementation occurred at 200 µg mL^−1^ [57]. The *upp* gene is widely used as a counterselection marker; however, the prerequisite for this approach is the deletion of *upp* gene from the genome of wild-type strain. In many bacterial species, such as *Clostridium acetobutylicum*, *C. glutamicum*,* Lactobacillus acidophilus*, *P. protegens*, the *upp* deletion does not affect the growth in control conditions, i.e., without 5-FU supplementation, but leads to increased 5-FU tolerance [57–60]. Here, we have used a newly developed genome modification system to delete *upp* gene in *B. methanolicus*. Similarly to other bacterial species, the *upp* deletion in *B. methanolicus* did not lead to a decrease in growth rate and biomass formation, and improved 5-FU tolerance in comparison to wild-type strain.

Altogether, we have tested and validated a functional counterselection system for *B. methanolicus* which enabled establishment of a novel method for genome modification in this bacterium. The deletion of *upp* was confirmed through PCR, genome sequencing, and physiological characterization the deletion strain.

## Supplementary Information

Supplementary Material 1.

## Data Availability

Whole genome sequencing raw data was submitted to the National Institute of Health (NIH) under BioProject ID PRJNA1259910.
