# Supplementary material for: Development of a markerless tool for targeted chromosome modification in the thermophilic and methylotrophic bacterium Bacillus methanolicus
Source: Microb Cell Fact. 2025 Dec 10;25:10. doi: 10.1186/s12934-025-02880-0 (PMC12802215; doi:10.1186/s12934-025-02880-0)
Supplement: Supplementary file 1 — Supplementary Material 1. [file 12934_2025_2880_MOESM1_ESM.pdf]

Supplementary material to

Development of a markerless tool for targeted chromosome modification in the thermophilic and methylotrophic bacterium *Bacillus methanolicus*

Marta Irla[1,2], Luciana Fernandes Brito[2], Jesper Langlo[2], Carsten Wohlers[3], Leonie Benninghaus[2,3], Chantel Heid[2], Volker F. Wendisch[3], Jochen Schmid[2,4], Trygve Brautaset[2*]

[1]Department of Biological and Chemical Engineering, Aarhus University, Gustav Wieds Vej 10D, Aarhus, Denmark

[2]Department of Biotechnology and Food Sciences, Norwegian University of Science and Technology, Sem Sælandsvei 6-8, NO-7491 Trondheim, Norway

[3]Genetics of Prokaryotes, Faculty of Biology and CeBiTec, Bielefeld University, Universitätsstraße 25, 33615 Bielefeld, Germany

[4]Institute of Molecular Microbiology and Biotechnology, University of Münster, Corrensstrasse 3, 48149 Münster, Germany

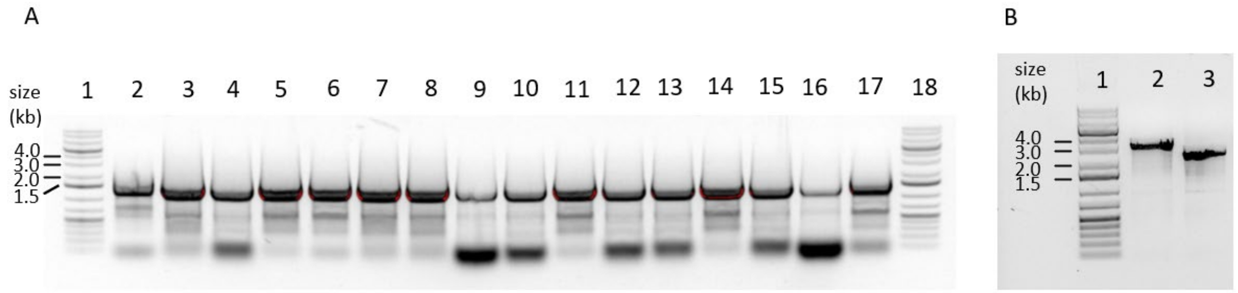

Figure S1. Colony PCR of chromosomal *upp* gene deletion in *B. methanolicus*. (A) Agarose gel electrophoresis picture of PCR products obtained with primers DEL29 and DEL30 using *B. methanolicus* colonies picked from the counterselection plate (lanes 2-17). Lanes 1 and 18 show the DNA ladder. (B) Agarose gel electrophoresis picture of PCR products obtained with primers DEL29 and DEL30 using chromosomal DNA of wild type strain (lane 2), and deletion strain (lane 3). Expected product sizes are 2813 bp and 2213 for lanes 2 and 3, respectively. Lane 1 shows the DNA ladder.

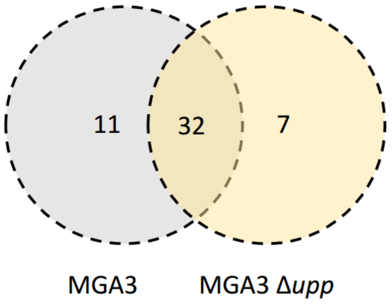

Figure S2. A study of genetic variants found in MGA3 and MGA3 Δ*upp* strains. The Venn diagram displays the shared and unique genetic variants detected in the genomes of MGA3 and MGA Δ*upp* strain in reference to the reference MGA3 genome sequenced in 2014 [1].

Table S1. Nucleotide variants determined by whole-genome sequencing. Unique variants occurring in the *B. methanolicus* strains MGA3 Δ*upp* in comparison to and to the the reference genome sequenced in 2014 [1]. POS -position of single-nucleotide polymorphism (SNP) in the genome; REF- the sequence in the reference genome; ALT-sequence in the mutant strain genome; MQ- mapping quality, EF- SNP effect; INDEL- small insertions and deletion; NR- non-redundant SNP; ncDNA-non-coding DNA.

Reference	POS	REF	ALT	MQ	DP	EF	Feature	Product
MGA3 Δ*upp*								
Chromosome	432,644	A	G	15	71	NR	BMMGA3_02225	hypothetical protein
	1,844,458	T	A	27	141	NR	BMMGA3_09230	hypothetical protein
	1,844,462	G	C	27	150	NR	BMMGA3_09230	hypothetical protein
	1,844,476	A	C	29	189	NR	BMMGA3_09230	hypothetical protein
	1,844,483	A	T	30	196	NR	BMMGA3_09230	hypothetical protein
	1,844,507	A	G	32	237	NR	BMMGA3_09230	hypothetical protein
	3,002,974	A	G	28	14	NR	ncDNA	ncDNA
MGA3								
Chromosome	453,536	T	C	23	132	NR	BMMGA3_02310	Spore cortex-lytic enzyme
	1,494,460	T	C	29	19	NR	BMMGA3_07525	hypothetical protein
	1,494,461	G	C	26	20	NR	BMMGA3_07525	hypothetical protein
	1,494,462	A	G	27	21	NR	BMMGA3_07525	hypothetical protein
	1,494,752	G	A	20	77	NR	BMMGA3_07525	hypothetical protein
	1,844,338	C	G	17	19	NR	BMMGA3_09225	hypothetical protein
	1,844,349	C	A	16	23	NR	BMMGA3_09225	hypothetical protein
	1,844,361	T	A	21	30	NR	BMMGA3_09225	hypothetical protein
	1,844,901	G	A	28	240	NR	ncDNA	ncDNA
	1,845,040	T	C	15	132	NR	ncDNA	ncDNA
	3,003,099	C	T	30	127	NR	ncDNA	ncDNA

1. Irla M, Neshat A, Winkler A, Albersmeier A, Heggeset TMB, Brautaset T, et al. Complete genome sequence of *Bacillus methanolicus* MGA3, a thermotolerant amino acid producing methylotroph. J Biotechnol 2014, 188, 110–111. [Google Scholar]
